# “Smart markets”: harnessing the potential of new technologies for endemic and emerging infectious disease surveillance in traditional food markets

**DOI:** 10.1128/jvi.01683-23

**Published:** 2024-01-16

**Authors:** Benjamin L. Sievers, Jurre Y. Siegers, Jimmy M. Cadènes, Sudipta Hyder, Frida E. Sparaciari, Filip Claes, Cadhla Firth, Paul F. Horwood, Erik A. Karlsson

**Affiliations:** 1 Virology Unit, Institut Pasteur du Cambodge, Phnom Penh, Cambodia; 2 Department of Medicine, University of Cambridge, Cambridge, United Kingdom; 3 Paris Institute of Technology for Life, Food and Environmental Sciences, AgroParisTech, Palaiseau, France; 4 Division of Infectious Disease, Columbia University Irving Medical Center, New York, New York, USA; 5 College of Public Health, Medical and Veterinary Sciences, James Cook University, Townsville, Queensland, Australia; 6 Emergency Centre for Transboundary Animal Diseases, Food and Agriculture Organization of the United Nations, Asia Pacific Region, Bangkok, Thailand; 7 EcoHealth Alliance, New York, New York, USA; 8 CANARIES: Consortium of Animal Networks to Assess Risk of Emerging Infectious Diseases through Enhanced Surveillance; Indiana University Bloomington, Bloomington, Indiana, USA

**Keywords:** viral surveillance, food markets, emerging infectious diseases, zoonotic diseases, wastewater, air sampling, smart markets, live bird markets, live animal markets, One Health

## Abstract

Emerging and endemic zoonotic diseases continue to threaten human and animal health, our social fabric, and the global economy. Zoonoses frequently emerge from congregate interfaces where multiple animal species and humans coexist, including farms and markets. Traditional food markets are widespread across the globe and create an interface where domestic and wild animals interact among themselves and with humans, increasing the risk of pathogen spillover. Despite decades of evidence linking markets to disease outbreaks across the world, there remains a striking lack of pathogen surveillance programs that can relay timely, cost-effective, and actionable information to decision-makers to protect human and animal health. However, the strategic incorporation of environmental surveillance systems in markets coupled with novel pathogen detection strategies can create an early warning system capable of alerting us to the risk of outbreaks before they happen. Here, we explore the concept of “smart” markets that utilize continuous surveillance systems to monitor the emergence of zoonotic pathogens with spillover potential.

**IMPORTANCE:**

Fast detection and rapid intervention are crucial to mitigate risks of pathogen emergence, spillover and spread—every second counts. However, comprehensive, active, longitudinal surveillance systems at high-risk interfaces that provide real-time data for action remain lacking. This paper proposes "smart market" systems harnessing cutting-edge tools and a range of sampling techniques, including wastewater and air collection, multiplex assays, and metagenomic sequencing. Coupled with robust response pathways, these systems could better enable Early Warning and bolster prevention efforts.

## INTRODUCTION

For most of history, humans worldwide have been separated by their language, customs, traditions, and culture. However, food distribution systems, as well as various chains of commerce that link them, connect people beyond borders. Traditional food markets (TFMs) are a mixture of these two factors, acting as vital trade sites and crucial gathering places for communities.

Food markets have received more global attention in recent years due to their association with the emergence and spread of several zoonotic pathogens. But what exactly is a TFM? From spices, vegetables, canned goods, and fruits to fresh and cured meats and sometimes even live animals (including both domestic and wild species), TFMs are places where people buy and sell a wide variety of goods. These markets can be found all around the world and come in many different shapes and sizes, including farmer’s markets, livestock auction yards, live wet markets, live animal markets, live bird markets (LBMs), and mixed markets.

With manifold benefits that range from providing nutrition and employment for communities to creating a gathering space for socializing and recreation, TFMs also carry infectious disease risks. These markets are social and economic gathering places that may contain foodstuffs, textiles, toys, tools, and potentially live animals in a contained environment. Pathogens that enter this system are provided an opportunity to amplify and spread, between one animal species and another or into people, including possible crossover into new host species (1). How can we mitigate this disease potential? It has been suggested that the complete closure of food markets that sell live animals may be a viable approach to decreasing the possibility of spillover events, yet shutting down markets can come with many unintended consequences.

Although temporary closures of food markets selling live animals have been shown to correlate with reduced risk of spillover, the permanent closure of all markets selling live animals remains highly controversial (2, 3). Increasing biosecurity in markets, including sequestration of animal species and separate slaughter points, can reduce risks but is often impractical or too costly in the given country contexts in least-developed countries (LDCs) (4, 5). Due to increasing urbanization, growing populations, and an expanding middle class in many LDCs, there is a growing demand for animal food products, especially protein (6). Food markets selling live animals are customary in many lower-resourced nations with limited cold chain infrastructure, as animals can be transported over considerable distances and housed for extended intervals without the need for refrigeration. Likewise, anecdotally, in many LDCs, people prefer to see live animals before purchasing, as the appearance of the animal can indicate the quality of the meat. Aside from providing income and food security for millions of people, food markets also carry considerable cultural significance for many communities and serve as hubs of economic and social activity (7). Although a global ban on food markets that sell live animals may not be a feasible solution to mitigating zoonotic disease emergence, reducing the risks posed by such markets is a feasible and worthy goal that may reduce the likelihood of future outbreaks and protect global public health.

To reduce disease risks associated with live animals and animal products in markets, a better understanding of pathogen circulation in these settings is of critical importance. Traditionally, market surveillance follows standard pathogen surveillance guidelines where teams enter markets, collect swabs from individual animals (along with environmental samples in some cases), and test them in a centralized facility with targeted molecular diagnostics such as real-time quantitative PCR (RT-qPCR). However, these surveillance programs are generally unable to provide a comprehensive picture of all pathogens circulating within a market due to a reliance on pre-identified pathogen targets. In addition, they require substantial cost, time, and risks to both the animals and the sampling team. How can we improve these systems to create a true “smart” market? Like the concept of a “smart city,” smart markets would gather data in near real-time at high-risk interfaces to increase biosecurity, and support early detection and rapid response systems (8).

### Environmental sampling of TFMs

Pathogens are everywhere: in the water, air, and the surfaces we interact with daily. Environmental sampling in TFMs provides vital insight into the presence, diversity, and possible transmission of pathogens between animals and humans while bypassing the need for sampling individual animals throughout the market. This method can streamline pathogen surveillance and provide a comprehensive understanding of the pathogens present in a TFM. It can also enhance knowledge of the various domestic, wildlife, and even vector species present in these markets.

#### Surface sampling

When visiting TFMs in Asia in the past, it was common to see local stall keepers rinsing and scraping the top layer of their wooden cutting boards throughout the day as the traditional board cleaning method. Nevertheless, the efficacy of this cleaning method has been disputed (9). Pathogen transmission occurs through both direct and indirect means, including skin-to-skin contact and the inhalation of dust particles (10). Some surfaces, such as chopping blocks that are not cleaned rigorously and used for slaughtering and butchering animals, may be ideal surfaces for sampling as they may contain potentially infectious material from many animals and may also serve as a source of contamination for meat products and humans. Pathogens can also remain on surfaces and in the environment for extended periods of time, creating unseen transmission risks. For example, severe acute respiratory syndrome coronavirus (SARS-CoV) and SARS-CoV-2 have viral viability for up to 72 hours on stainless steel (11). Similarly, several influenza virus strains exhibit prolonged persistence in the environment, with viable virus recovered after 2 weeks in/on some substrates (12, 13). As both highly pathogenic avian influenza and low pathogenic avian influenza viruses are of critical concern globally, this tendency towards environmental persistence is of particular concern in the context of TFMs. Understanding the persistence and prevalence of pathogens on different surfaces is paramount for understanding potential transmission within TFMs.

Many protocols exist for surface sampling. While they can vary in methodology depending on the targeted pathogen, they also follow similar procedures. The tip of a sterile swab is dragged vertically and horizontally across a surface and quickly submerged in preservation media. At a laboratory or other testing facility, this sample can then be used for a myriad of tests depending on the pathogen type, although molecular diagnostics are increasingly dominant (14, 15). Since many important zoonotic pathogens exhibit significant environmental persistence, the inclusion of surface sampling can bolster the probability of detection for many pathogens of concern, including respiratory viruses. Fortunately, many systems, including RT-PCR and next-generation sequencing, exist to detect miniscule amounts of pathogen genetic material, increasing the likelihood of detecting a pathogen from a swab. Environmental surface sampling can give insight into pathogens present on a range of surfaces in TFMs, allowing the development of targeted interventions to reduce risks associated with environmental persistence and aid in enhancing biosecurity measures.

#### Wastewater and wash water

While wastewater surveillance surged into the public limelight during the coronavirus disease 2019 (COVID-19) pandemic, these techniques first gained traction in the 1990s to aid in the elimination of poliovirus. As polio circulation continued in several countries such as Egypt, Israel, Pakistan, India, and Nigeria, the Global Polio Eradication Initiative developed systems to test wastewater that were four to five times more sensitive than the other methods for poliovirus detection used in communities (16, 17). Similarly, wastewater testing for SARS-CoV-2 has revealed the power of passive monitoring to contribute to the rapid detection and tracking of new variants/strains of concern while providing insight into the epidemiology of a rapidly emerging pathogen within an unprecedented timeframe. With great success in passively monitoring SARS-CoV-2 prevalence in communities during the COVID-19 pandemic, wastewater testing has proven to be an effective method for inferring transmission dynamics and tracking outbreaks (18).

**Fig 1:**
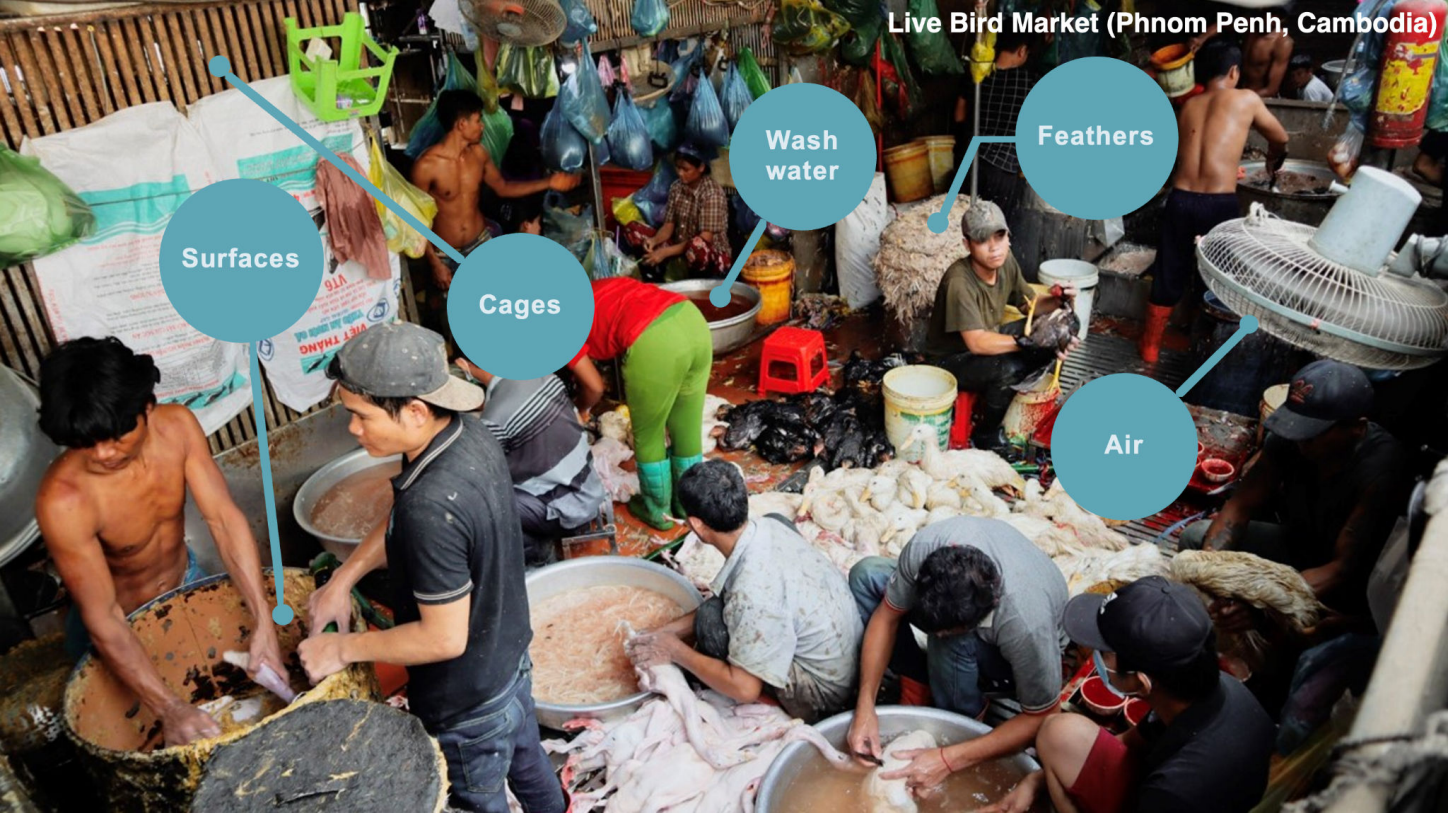
Orussey Live Bird Market, Phnom Penh, Cambodia. Photo courtesy of Erik A. Karlsson from Virology Unit, Institut Pasteur du Cambodge.

Extending beyond poliovirus and SARS-CoV-2, the benefits of using wastewater surveillance in TFMs are broad-ranging. Every day as LBMs prepare for closure, shop owners rinse the floors and cages, allowing the water runoff to flow into drainage systems (Fig. 1). Sampling LBM wastewater can give insight into both the pathogens and the species in the LBM without the need for sampling individual animals, people, or even surfaces. Wastewater testing relies on the presence of pathogen material in the bodily fluids and other material washed into the drainage system. Therefore, it is perhaps best suited to detecting enteric pathogens; however, respiratory infections and even some arboviruses can be detected. For example, human influenza A viruses (IAVs), which are associated with endemic seasonal influenza, have high rates of fecal shedding, and some infected individuals display fecal titers longer than nasal titers, making IAV a potential target for wastewater screening (19, 20). It has also recently been demonstrated that some vector-borne arboviruses can be detected in untreated wastewater samples, including dengue, Zika, and yellow fever viruses (21, 22). Similar to wastewater, carcass wash water may also be an ideal environmental sample type for the detection of viral pathogens in slaughtering areas of TFMs. In places like swine abattoirs and poultry slaughtering areas of LBMs, animal carcasses are thoroughly rinsed with water to remove the bodily fluids and excreta that result from the butchering process (23, 24). This wash water is often used for multiple carcasses and could prove to be a comprehensive sample type for pathogen surveillance across a whole market.

Wastewater samples can be taken routinely through manual, passive, or automated systems to monitor pathogens in TFMs. There are several commercial wastewater samplers in use for active surveillance, and each method varies in the sampling and detection processes. For example, using wastewater to forecast a 17-day citywide flu outbreak in Ottawa, Canada, Mercier et al. (19) were able to quantify, type, and subtype influenza virus from primary sludge, and municipal wastewater manually collected, clarified, and analyzed through RT-qPCR. Using a different method than the previous group, a cluster of COVID-19 cases was detected in an apartment building in Singapore through wastewater sampling, where Wong et al. (25) utilized an autosampler (Aquacell P2 Multiform, Aquamatic, United Kingdom) to draw wastewater samples from a manhole for RNA extraction and RT-qPCR. Looking ahead to the future, environmental pathogen surveillance may follow the trend in wearable technology, as the eDNA Sampler from Smith-Root resembles a backpack yet contains a fully integrated environmental DNA sampling system with a computer-controlled pump to draw in and directly capture environmental DNA. Despite the promise of this technology, more experimentation will be required for virus detection (26).

Wastewater/wash water sampling can occur using a variety of collection, concentration, and treatment methods; however, almost every method follows a similar series of steps. First, wastewater or primary sludge is collected manually or through a pump system and exposed to various levels of treatment, from no treatment to thermal deactivation. The wastewater product can then be concentrated through a number of methods, including ultracentrifugation, filtration, and precipitation. Following nucleic acid extraction, most methods will then use RT-qPCR for virus detection and quantification (27). By employing comprehensive RT-qPCR or consensus PCR panels that leverage highly conserved regions of pathogen genomes for detection, wastewater samples can be used to monitor viruses circulating in TFMs and aid in the discovery of new viruses. Importantly, innovation in this approach can aid in the development of systems that can detect novel pathogens from commensals within the environment. While wastewater testing has found considerable success in many developed nations in various contexts, the development of a standardized wastewater testing pipeline for markets that could be easily deployed in LDCs will be important for the success of this sample type.

#### Air sampling

Similar to wastewater, air sampling has recently gained traction during the COVID-19 pandemic as an approach for detecting and monitoring pathogens in communal spaces. However, the invention of air sampling dates back to the 1860s. Early air samplers, known as impactors, were used mainly to study the relationship between dust and disease, utilizing small jets of air impinging on a plate enabling aerosolized material to accumulate for further analysis, forming the basis of modern-day air sampling (28). Air sampling could provide an ideal system for pathogen surveillance in TFMs. As a surveillance tool, air sampling has the capacity to provide early detection signals for pathogens of concern, enabling timely intervention and rapid response. For example, in 2014–2015, air sampling in live poultry markets in China successfully resulted in the isolation of A/H5N6, A/H7N9, and A/H9N2 avian influenza viruses, enabling swift intervention (29). The broad coverage provided by air sampling in TFMs will likely increase the frequency at which pathogens are detected, improving preparedness and potentially contributing to future vaccination design. Expanding upon the method explored by Zhoe et al. (29), we envision strategically placing air samplers throughout TFMs to serve as sentinels that can provide both virus detection and specific information on the distribution and prevalence of pathogens throughout the market.

**Fig 2:**
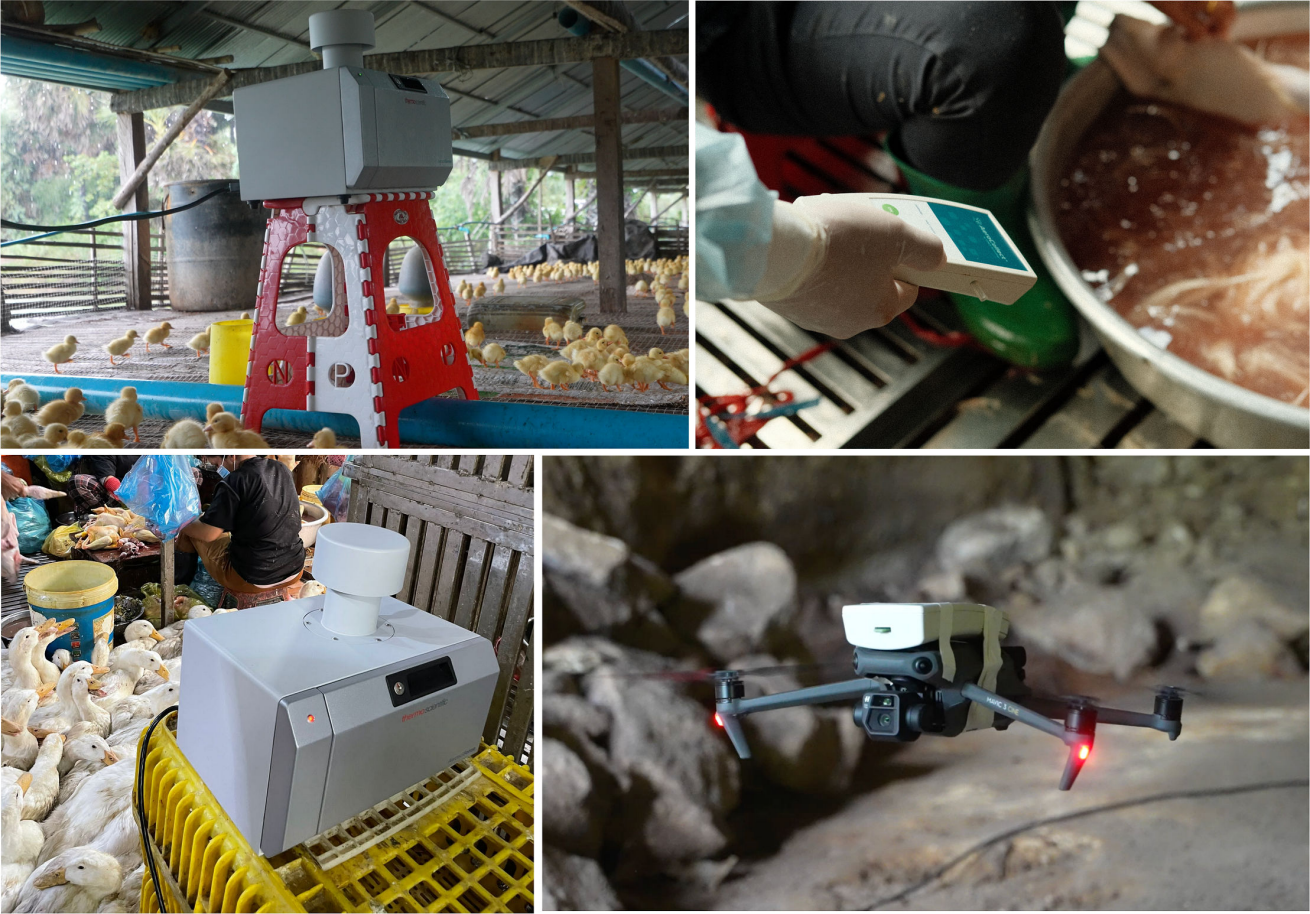
Air sampling devices can vary in size, shape, and detection mechanism. Photos courtesy of Benjamin L. Sievers and Jurre Y. Siegers from Virology Unit, Institut Pasteur du Cambodge.

Many types of air samplers available for purchase vary in size and collection method and are envisioned to play different roles in TFMs (Fig. 2). Acting as silent observers of the air, wearable air samplers can capture pathogens from the ambient air around a TFM worker giving a more representative look into individual pathogen exposure. In one study conducted in Phnom Penh, Cambodia, an air sampling unit modified to operate at a flow rate simulating normal human breathing was deployed in an LBM leading to the isolation of IAVs H5N1 and H9N2 (30). Similarly, a group in Guangdong, China, utilized a wearable air sampler and successfully detected H7 IAV in market air (31). Larger, more powerful air samplers are envisioned to be placed in areas with significant human and animal density, screening larger amounts of ambient air in TFMs.

**Fig 3:**
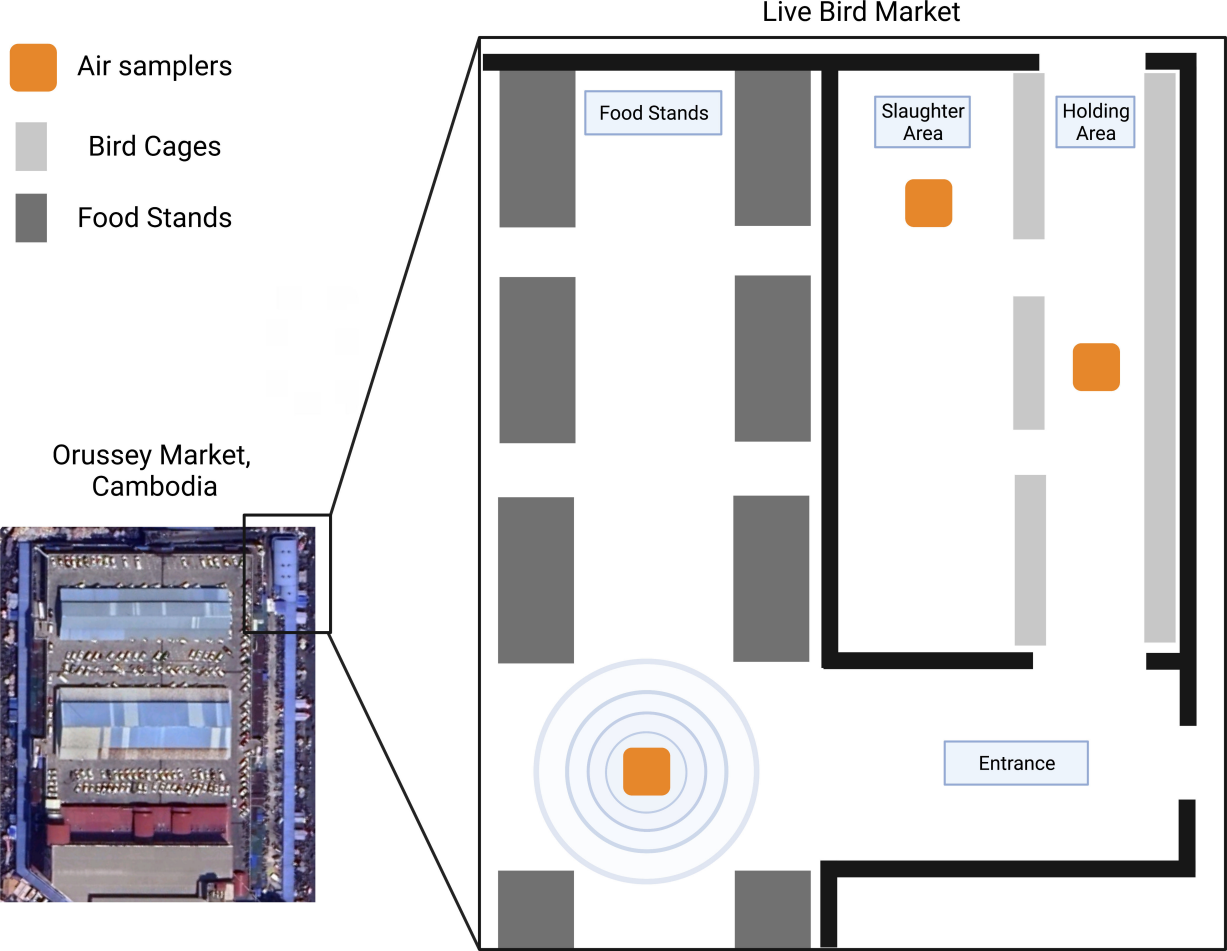
Floor plan of Orussey Live Bird Market in Phnom Penh, Cambodia, with approximate air sampler placement highlighted in orange.

Most air sampling methods rely on capturing pathogen genetic material or virions from ambient air on a membrane or cartridge for further analysis. Turning on the sampler causes air to flow through a specialized filter or chip-based sample chamber where virions and viral genetic material accumulate. The viral material is eluted or removed from the filter for detection or, in some cases, culture. In ideal circumstances, air samplers would be strategically implemented, enabling precise location data within the market for virus detection (Fig. 3). There are manifold benefits to continuous air sampling in TFMs, as it represents a non-invasive, relatively inexpensive method that can be used for the collection and detection of many emerging and re-emerging pathogens.

### So now we have a comprehensive sample: what do we do with it?

#### Marketside testing

In concert with these new sampling methods, we can reduce the time to results even further using one of the numerous portable field-ready technologies now making rapid “marketside” testing feasible. From portable gel electrophoresis systems to portable nucleic acid sequencers, the possibility of complete field sampling to detection has been realized. Once we have acquired a comprehensive surveillance sample from a TFM, the next step is processing and analyzing the sample for pathogens and/or host identification. This era of technological advancement has ushered in a new wave of technology that can now streamline sample analysis. Complete field-ready PCR thermocyclers, and even qPCR thermocyclers, are now available for purchase and deployment, enabling real-time pathogen detection on-site between the busy vendor booths of TFMs. Portable sequencers, such as the MinION from Oxford Nanopore Technologies, also bring detection market side as total DNA and RNA sequencing can commence outside of the laboratory. As the number of TFMs dramatically exceeds the number of facilities capable of pathogen testing, these market-side testing technologies can streamline sample analysis, especially in places most distant from laboratories, significantly decreasing outbreak and routine surveillance response time.

#### Metagenomic sequencing

With significant advancements in molecular biology, bioinformatics, and raw computing power in the 1990s–2000s, metagenomics has proven to be a very promising tool for studying microbial communities in the environment (32–34). In recent years, metagenomics has significantly advanced through the development of metagenomic next-generation sequencing (mNGS) and third-generation sequencing techniques, as we can now study “all” genetic materials present in a sample across a diverse range of samples, with strain-resolved resolution and high sensitivity, commonly known as “shotgun metagenomics” (35, 36). Recent advancements in mNGS have rendered this technique a compelling approach for both pathogen surveillance and pathogen discovery in TFMs (37). A technique known as viral metatranscriptomics now enables the generation of whole genomes for all RNA viruses within a given sample in an unbiased nature (38). This technique carries the massive potential to aid in the discovery and characterization of viruses within a given sample from a TFM, as it is nonspecific, enabling the identification of known and unknown pathogens from sequence data alone.

Of the methods previously described, metagenomics and shotgun metagenomics are highly informative methods for pathogen surveillance and identification in TFMs (39). There are two leading platforms for shotgun metagenomics for pathogen surveillance in TFMs: short-read sequencing (e.g., Illumina) and long-read sequencing (e.g., Oxford Nanopore). The short-read platform delivers high sequencing depth and accuracy but has processing times of up to several days (40, 41). Long-read platforms are more rapid, enabling portable *de novo* assembly capabilities ideal for quick field deployment and intervention in TFMs yet offer less sequencing depth and accuracy. Combined, deploying both of these strategies in TFMs can yield great benefits, as short-read platforms can provide robust, cost-effective metagenomic information for routine TFM pathogen surveillance, while long-read platforms can be deployed in outbreak settings requiring speed. Aside from unbiased shotgun metagenomics, targeted metagenomic methods can also bring increased specificity into pathogen surveillance in TFMs, as these methods can be used to identify pathogens with increasingly similar genomes.

Despite some of the challenges associated with metagenomics, such as cost, technical expertise required, and the need for standardized workflows, continuous environmental sampling paired with metagenomics holds significant potential to bring a clearer vision of the pathogens present within TFMs. We envision metagenomics bringing a deeper layer of surveillance to TFMs in both continuous monitoring and outbreak settings.

#### Pathogen agnostic testing

##### Thermal sensor-based surveillance

With a thin blackened strip of platinum, a Wheatstone bridge circuit, and an ammeter, an American astronomer, Samuel Langley, made a device capable of measuring a cow’s heat from ~400 m away (42). Nearly 50 years later, Langley’s early invention was built upon, as Hungarian physicist Kálmán Tihanyi developed the evaporograph, an infrared-sensitive electronic television camera for anti-aircraft defense declassified by Britain in 1956 (43). Today, thermal sensor-based surveillance systems enable rapid detection of actively febrile individuals. Most commonly seen in airports, grocery stores, and other high pedestrian traffic areas during the COVID-19 pandemic, thermal image scanning is a relatively high-throughput surveillance system to find feverish individuals. Because fever is a broad symptom associated with many infectious diseases, prompt recognition of febrile individuals may allow intervention and delay the importation of infection into other environments. Notably, most animals and nearly all mammals can develop fevers when infected with an infectious pathogen (44). In a proof-of-concept study, using thermal imaging cameras, researchers could differentiate between febrile birds infected with highly pathogenic avian influenza and uninfected birds within a flock in a small chicken pen (45). While preliminary research looks promising, further research is needed to establish thermal imaging as a robust surveillance technique.

As the benefits of thermal sensing systems continue beyond monitoring the animals of the market, thermal sensing can also be utilized in TFMs in places with high pedestrian congestion. In addition to surveilling congregate animal-holding areas, thermal sensing systems can delay the importation of disease from humans back into animals. Infrared thermal detection systems for mass fever screening in hospitals have successfully detected febrile individuals in real time with high sensitivity and specificity, making thermal screening implementation in TFMs potentially feasible for animals and humans (46). Conceivably, utilizing advancements in machine learning, a thermal sensing system could be paired with artificial intelligence capable of identifying and quantifying spikes in the number of febrile individuals, thereby activating an alert system to the potential of a developing outbreak.

##### Acoustic monitoring

Through the detection of coughing, sneezing, grunting, or even silence, recent developments in bioacoustic monitoring devices enable ecologists and epidemiologists to use sound to characterize the behaviors, spatial estimation, occupancy, and general health of wildlife species (47, 48). Coughing, consistent with respiratory disease or distress, is an excellent marker for bioacoustic monitoring as coughing manifests as a characteristic and distinctive soundwave sufficiently different from other noises. In one study conducted in Jinju, Korea, by Chung et al. (49), researchers were able to employ bioacoustic monitoring and a simple algorithm to separate acoustic footprints of coughing noises from background noise to accurately identify general areas with sick animals in a pig breeding farm. While bioacoustic monitoring may not be able to provide us with specific information on the identity of the pathogens in circulation, they do provide early warning signals that sickness may be spreading throughout a population. In this way, automation of acoustic monitoring may provide early warning signals prompting timely biosecurity intervention and aiding in the continuous surveillance of TFMs for pathogens. As with any surveillance system that captures human data, public trust and the development of robust systems to ensure data privacy and security will be critical for the success of any surveillance.

### Putting it all together - the future of “smart” markets

Surveillance technology is developing and progressing at phenomenal speeds. The futuristic cities imagined in science fiction are closer than expected. The concept of “smart” systems is not new by any means. “Smart cities” are proposed environments capable of collecting and parsing large amounts of population data through surveillance devices and communication networks and have existed in literature for decades (50). These systems streamline surveillance with automation and early detection mechanisms. Although the formation of smart surveillance systems is largely controversial due to privacy concerns, the potential benefits of applications in the medical, public health, environmental, and animal health sectors are exceptional.

The ideal smart market would harness the power of continuous surveillance technology for safeguarding health and preventing outbreaks in TFMs. By strategically integrating specific and nonspecific host and pathogen surveillance systems, such as wastewater and air sampling, with approaches such as metagenomic sequencing and acoustic monitoring, this model provides a unique advanced warning system using real-time data collection and analysis.

Imagine entering a bustling smart market filled with families, vendors, animals, and food. As you step into this lively ecosystem, passive pathogen surveillance technology seamlessly blends into the surroundings, collecting continuous pathogen data to keep us safe. Air sampling machines hum quietly, well positioned in the environment, capturing airborne particles from the air. Wastewater sampling devices sit patiently and discreetly at drainage sites from the animal stalls, continuously screening for pathogens and offering insights into chicken health. Overhead, bioacoustic sensors resembling speakers are placed strategically to listen to the animals throughout the night. This network of continuous surveillance systems creates a dynamic interwoven mosaic, enabling swift and comprehensive pathogen surveillance of TFMs.

**Fig 4:**
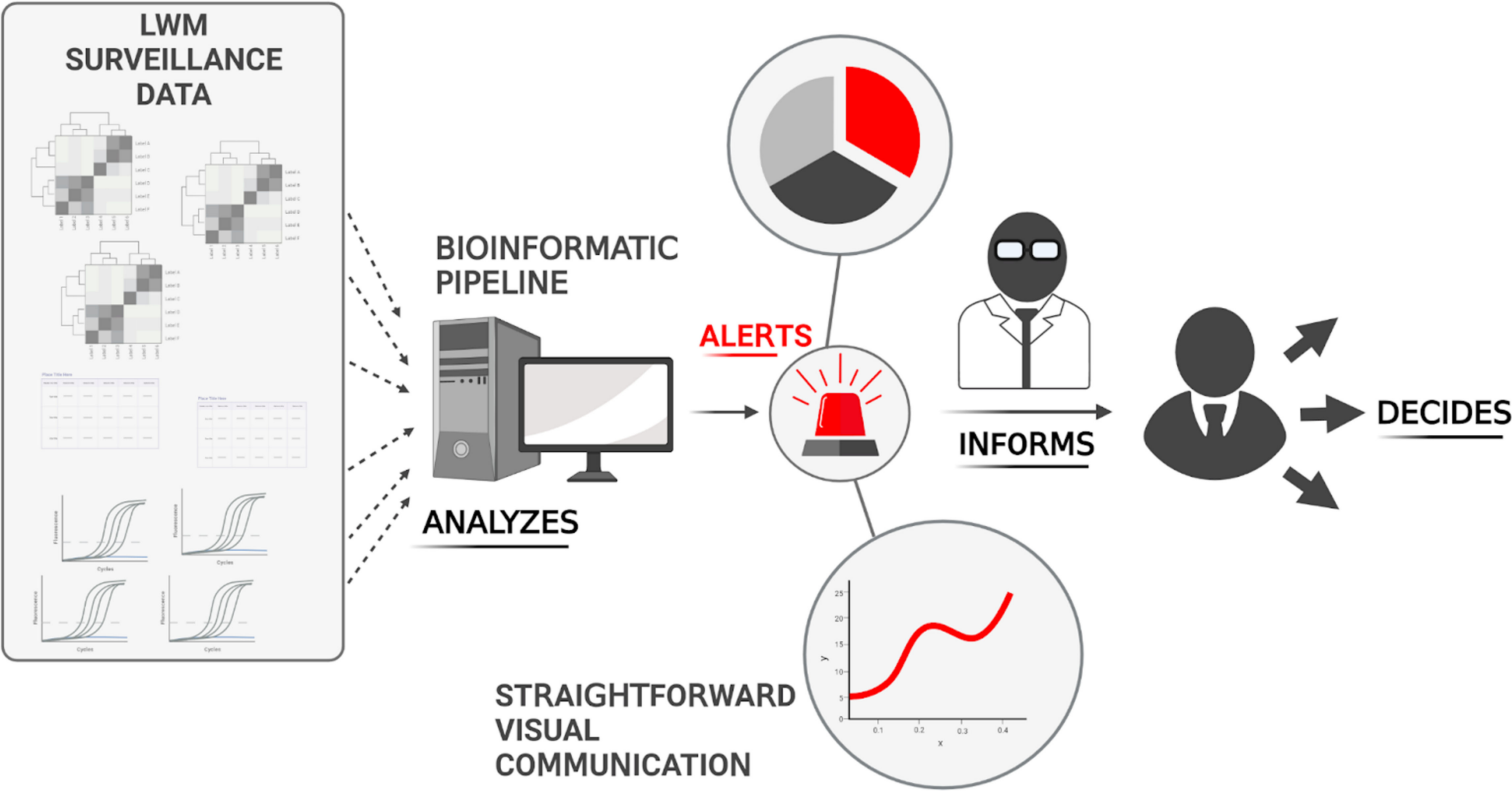
Integration of bioinformatic pipelines to transform raw data into straightforward information for scientists and decision-makers.

**Fig 5:**
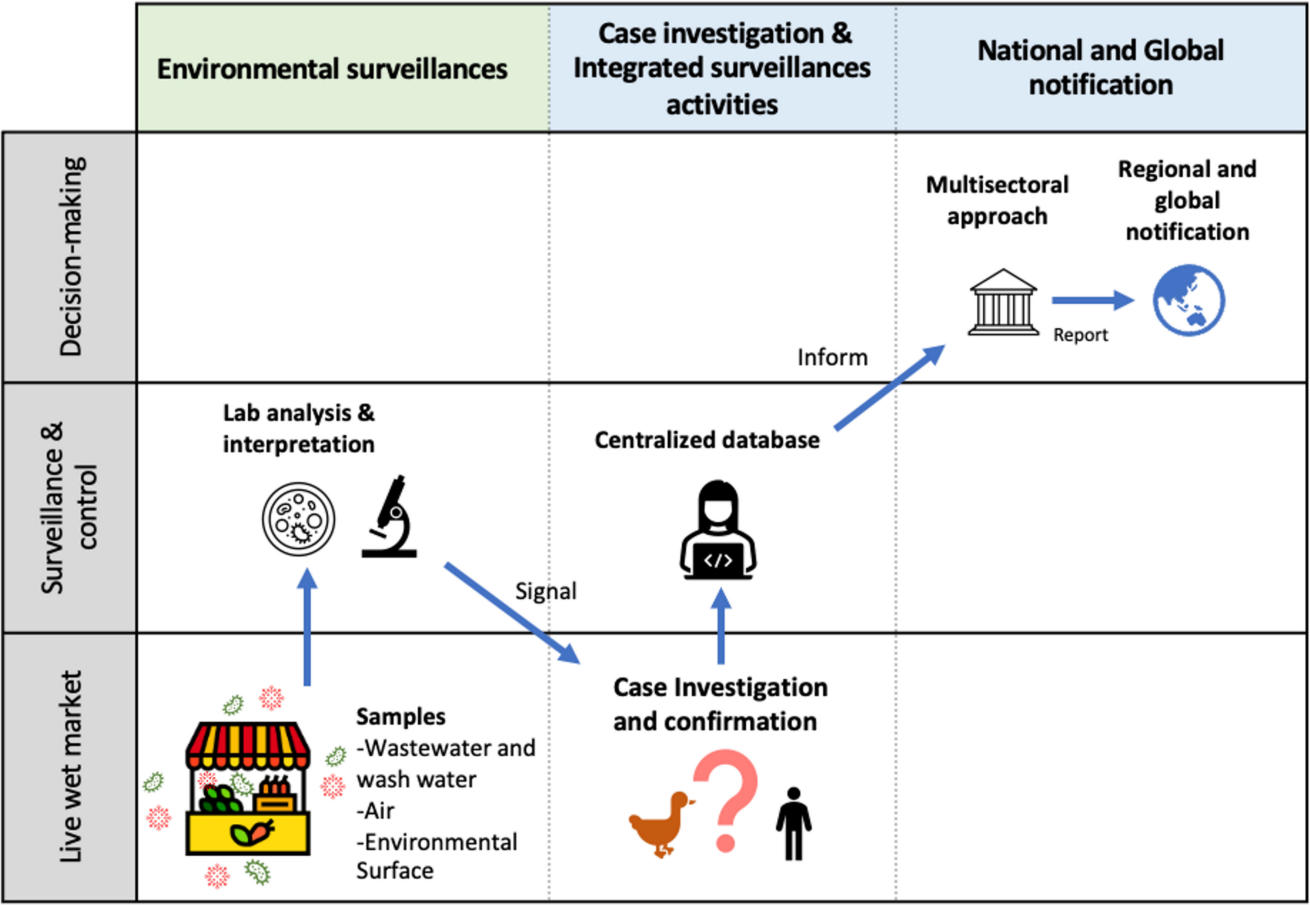
Prospective pathway for reporting pathogens of concern for environmental samples.

In practice, robust surveillance systems are most effective with thoughtful and productive intervention and reporting plans. When a pathogen of high concern is detected through environmental sampling and confirmed in a laboratory setting, a response team can be initiated to conduct rapid, targeted sampling triage in the affected area of the TFM. Without causing unnecessary alarm, an intervention team can find the affected site, isolate the animals, and work with exposed individuals. The highest success of this approach is foreseen in communities with non-endemic emerging infectious diseases (EIDs), as the system can enable a rapidly targeted response to emerging pathogens. Using bioinformatic pipelines and metagenomics can significantly enhance the speed and efficiency of tracking and detecting emerging viral threats. Through the analysis of the vast amount of data produced by continuous environmental sampling, these pipelines have the potential to condense and analyze information previously impossible without modern computing power (Fig. 4). In communities where EIDs are endemic and occur at a higher rate in TFMs, the smart market system can aid in outlining and assembling a more comprehensive impression of viral prevalence within a community. Following an initial case investigation or routine surveillance, the information on the incident can be shared throughout national and global networks, enabling a multisectoral response (Fig. 5).

As each country differs in the funding, personnel, and ability to implement surveillance technology, the ideal environmental surveillance system for TFMs would be consistent, inexpensive, and easily reproducible. To achieve this, a standardized protocol is envisioned utilizing a list of affordable and durable equipment and employing a periodical systemized sampling schedule. With increased accessibility to surveillance technology, surveillance strategies could prioritize communities that serve as hubs for rural/remote areas, as people in these communities may have frequent contact with wild and domestic animals, with increasing zoonotic disease exposure risks (51).

With the data collected from continuous pathogen surveillance, a risk category classification system for TFMs could be developed to increase biosecurity, biosafety, and target intervention methods to keep markets safe. This system could be used to help develop and evaluate other methods of mitigating spillover risk, such as improved segregation of animals and pathogen screening protocols. Likewise, as there is an increase in metagenomic information acquired from TFMs, the development and implementation of a metagenomic viral surveillance matrix are envisioned to outline details on viral movement between livestock and humans (52). To aid in the visualization and parsing of the vast amount of data generated from the metagenomic analysis of environmental samples, we envision the use of advanced bioinformatic pipelines capable of analyzing large data sets and producing clean and easily visualized summary reports (53–58). Importantly, the relative speed of these surveillance pipelines is only as fast as their rate-limiting step. Ongoing efforts to streamline potentially slower steps such as PCR and sequencing can further assist to bypass bottlenecks and expedite turnaround. Appropriate data safety protocols must be implemented to ensure data sharing and availability, while protecting the human right to privacy.

Smart markets employing continuous environmental surveillance have the potential to intercept spillover events before they happen. Through meticulous categorization of known pathogens, one study noted that 60.3% of EIDs in humans were of zoonotic origin and nearly 72% of those originated in wildlife (59). As a common gathering place for wild animals, domestic animals, and humans, TFMs represent an interface that carries a significant spillover risk. With this intensity of interaction, there is a high likelihood that future pandemics will arise from TFMs.

A One Health approach that highlights the integrated nature of humans, animals, and the environment is widely acknowledged as the most comprehensive and effective approach to address emerging zoonotic pathogens (60). One Health involves multiple sectors, disciplines, and communities at varying levels of society working to foster well-being and tackling threats to human, animal, and environmental health. To maximize the benefits of a continuous environmental surveillance program, a multidisciplinary, multisectoral approach is necessary. Successful implementation relies on collaboration between key stakeholders, including global, local, and regional entities. Implementing surveillance systems in these TFMs can carry manifold benefits, from an early warning system for emerging diseases to further documenting the prevalence of viruses in animal communities (8, 61).

### Conclusion

To prevent the next pandemic, a combinatorial One Health approach is warranted. Drastic changes to the food supply system, such as shutting down food markets that sell animals, have been shown to be ineffective (2). Instead, rapid data sharing and availability will be vital to any successful surveillance and intervention program. Continuous environmental surveillance is a tool with significant potential to delay future spillover events; however, technology cannot prevent outbreaks alone. With this in mind, outbreak/pandemic prevention through the development and implementation of robust biosecurity and biosafety measures responsive to consistent pathogen surveillance results should become the main focus of EID management. Importantly, smart market systems can stand as a marker or tool to assess the impact of biosecurity interventions on viral diversity in food markets, such as better air circulation, using lids on defeathering machines, and separating slaughter areas from holding areas.

Currently, mass implementation of surveillance systems such as wastewater testing and air sampling in TFMs remains challenging. Implementing these systems often comes with high costs (both implementation and upkeep) that are unreasonable for many LDCs. Innovations and benchmarking are needed to overcome the monetary and logistical challenges associated with environmental sampling; however, these systems will transform how we sample pathogens today. Innovative or Orwellian, regardless, smart surveillance systems in TFMs carry immense potential to prevent future spillover events, potentially stopping pandemics before they happen. An ounce of prevention is worth a pound of cure.

## References

[1] Galindo-González J. 2022. Live animal markets: Identifying the origins of emerging infectious diseases. Curr Opin Environ Sci Health 25:100310. doi:10.1016/j.coesh.2021.100310 34931177 PMC8674032

[2] Li Y, Wang Y, Shen C, Huang J, Kang J, Huang B, Guo F, Edwards J. 2018. Closure of live bird markets leads to the spread of H7N9 influenza in China. PLoS One 13:e0208884. doi:10.1371/journal.pone.0208884 30540847 PMC6291110

[3] Shi N, Huang J, Zhang X, Bao C, Yue N, Wang Q, Cui T, Zheng M, Huo X, Jin H. 2020. Interventions in live poultry markets for the control of avian influenza: a systematic review and meta-analysis. J Infect Dis 221:553–560. doi:10.1093/infdis/jiz662 31323094

[4] FAO. 2015. Biosecurity guide for live poultry markets

[5] FAO animal production and health guidelines No.17. Rome, Italy.

[6] Abu Hatab A, Cavinato MER, Lagerkvist CJ. 2019. Urbanization, livestock systems and food security in developing countries: a systematic review of the literature. Food Sec 11:279–299. doi:10.1007/s12571-019-00906-1

[7] Shanbehzadeh M, Nopour R, Kazemi-Arpanahi H. 2022. Designing a standardized framework for data integration between zoonotic diseases systems: towards one health surveillance. Inform Med Unlocked 30:100893. doi:10.1016/j.imu.2022.100893

[8] Keusch GT, Amuasi JH, Anderson DE, Daszak P, Eckerle I, Field H, Koopmans M, Lam SK, Das Neves CG, Peiris M, Perlman S, Wacharapluesadee S, Yadana S, Saif L. 2022. Pandemic origins and a One Health approach to preparedness and prevention: solutions based on SARS-CoV-2 and other RNA viruses. Proc Natl Acad Sci U S A 119:e2202871119. doi:10.1073/pnas.2202871119 36215506 PMC9586299

[9] Lo MY, Ngan WY, Tsun SM, Hsing H-L, Lau KT, Hung HP, Chan SL, Lai YY, Yao Y, Pu Y, Habimana O. 2019. A field study into Hong Kong's wet markets: raised questions into the hygienic maintenance of meat contact surfaces and the dissemination of microorganisms associated with nosocomial infections. Front Microbiol 10:2618. doi:10.3389/fmicb.2019.02618 31781084 PMC6861454

[10] Louten J. 2016. Virus transmission and epidemiology. Essent Hum Virol:71–92. doi:10.1016/B978-0-12-800947-5.00005-3

[11] van Doremalen N, Bushmaker T, Morris DH, Holbrook MG, Gamble A, Williamson BN, Tamin A, Harcourt JL, Thornburg NJ, Gerber SI, Lloyd-Smith JO, de Wit E, Munster VJ. 2020. Aerosol and surface stability of SARS-CoV-2 as compared with SARS-CoV-1. N Engl J Med 382:1564–1567. doi:10.1056/NEJMc2004973 32182409 PMC7121658

[12] Thompson K-A, Bennett AM. 2017. Persistence of influenza on surfaces. J Hosp Infect 95:194–199. doi:10.1016/j.jhin.2016.12.003 28139390

[13] Brown JD, Swayne DE, Cooper RJ, Burns RE, Stallknecht DE. 2007. Persistence of H5 and H7 avian influenza viruses in water. Avian Dis 51:285–289. doi:10.1637/7636-042806R.1 17494568

[14] Sanderson WT, Hein MJ, Taylor L, Curwin BD, Kinnes GM, Seitz TA, Popovic T, Holmes HT, Kellum ME, McAllister SK, Whaley DN, Tupin EA, Walker T, Freed JA, Small DS, Klusaritz B, Bridges JH. 2002. Surface sampling methods for Bacillus anthracis spore contamination. Emerg Infect Dis 8:1145–1151. doi:10.3201/eid0810.020382 12396930 PMC2730285

[15] Julian TR, Tamayo FJ, Leckie JO, Boehm AB. 2011. Comparison of surface sampling methods for virus recovery from fomites. Appl Environ Microbiol 77:6918–6925. doi:10.1128/AEM.05709-11 21821742 PMC3187074

[16] Asghar H, Diop OM, Weldegebriel G, Malik F, Shetty S, El Bassioni L, Akande AO, Al Maamoun E, Zaidi S, Adeniji AJ, Burns CC, Deshpande J, Oberste MS, Lowther SA. 2014. Environmental surveillance for polioviruses in the global polio eradication initiative. J Infect Dis 210:S294–S303. doi:10.1093/infdis/jiu384 25316848 PMC10578309

[17] Kroiss SJ, Ahmadzai M, Ahmed J, Alam MM, Chabot-Couture G, Famulare M, Mahamud A, McCarthy KA, Mercer LD, Muhammad S, Safdar RM, Sharif S, Shaukat S, Shukla H, Lyons H. 2018. Assessing the sensitivity of the polio environmental surveillance system. PLOS ONE 13:e0208336. doi:10.1371/journal.pone.0208336 30592720 PMC6310268

[18] Larsen DA, Wigginton KR. 2020. Tracking COVID-19 with wastewater. Nat Biotechnol 38:1151–1153. doi:10.1038/s41587-020-0690-1 32958959 PMC7505213

[19] Mercier E, D’Aoust PM, Thakali O, Hegazy N, Jia J-J, Zhang Z, Eid W, Plaza-Diaz J, Kabir MP, Fang W, Cowan A, Stephenson SE, Pisharody L, MacKenzie AE, Graber TE, Wan S, Delatolla R. 2022. Municipal and neighbourhood level wastewater surveillance and subtyping of an influenza virus outbreak. Sci Rep 12:15777. doi:10.1038/s41598-022-20076-z 36138059 PMC9493155

[20] Al Khatib HA, Coyle PV, Al Maslamani MA, Al Thani AA, Pathan SA, Yassine HM. 2021. Molecular and biological characterization of influenza A viruses isolated from human fecal samples. Infect Genet Evol 93:104972. doi:10.1016/j.meegid.2021.104972 34153546

[21] Lee WL, Gu X, Armas F, Leifels M, Wu F, Chandra F, Chua FJD, Syenina A, Chen H, Cheng D, Ooi EE, Wuertz S, Alm EJ, Thompson J. 2022. Monitoring human arboviral diseases through wastewater surveillance: challenges, progress and future opportunities. Water Res 223:118904. doi:10.1016/j.watres.2022.118904 36007397

[22] Chandra F, Lee WL, Armas F, Leifels M, Gu X, Chen H, Wuertz S, Alm EJ, Thompson J. 2021. Persistence of dengue (serotypes 2 and 3), zika, yellow fever, and murine hepatitis virus RNA in untreated wastewater. Environ Sci Technol Lett 8:785–791. doi:10.1021/acs.estlett.1c00517

[23] Horm SV, Tarantola A, Rith S, Ly S, Gambaretti J, Duong V, Y P, Sorn S, Holl D, Allal L, Kalpravidh W, Dussart P, Horwood PF, Buchy P. 2016. Intense circulation of A/H5N1 and other avian influenza viruses in Cambodian live bird markets with serological evidence of sub-clinical human infections. Emerging Microbes & Infections 5:1–9. doi:10.1038/emi.2016.69 PMC5141262 27436362

[24] Horwood PF, Horm SV, Suttie A, Thet S, Rith S, Sorn S, Holl D, Tum S, Ly S, Karlsson E, Tarantola A, Dussart P. 2018. Co-circulation of influenza A/H5N1 with H7 and H9 viruses in Cambodian live bird markets with evidence of frequent co-infections in poultry. Emerging Infect Dis 24:352–355. doi:10.3201/eid2402.171360 PMC5782910 29350140

[25] Wong JCC, Tan J, Lim YX, Arivalan S, Hapuarachchi HC, Mailepessov D, Griffiths J, Jayarajah P, Setoh YX, Tien WP, Low SL, Koo C, Yenamandra SP, Kong M, Lee VJM, Ng LC. 2021. Non-intrusive wastewater surveillance for monitoring of a residential building for COVID-19 cases. Sci Total Environ 786:147419. doi:10.1016/j.scitotenv.2021.147419 33964781 PMC8081581

[26] Thomas AC, Howard J, Nguyen PL, Seimon TA, Goldberg CS, Golding N. 2018. eDNA sampler: a fully integrated environmental DNA sampling system. Methods Ecol Evol 9:1379–1385. doi:10.1111/2041-210X.12994

[27] Abdeldayem OM, Dabbish AM, Habashy MM, Mostafa MK, Elhefnawy M, Amin L, Al-Sakkari EG, Ragab A, Rene ER. 2022. Viral outbreaks detection and surveillance using wastewater-based epidemiology, viral air sampling, and machine learning techniques: a comprehensive review and outlook. Sci Total Environ 803:149834. doi:10.1016/j.scitotenv.2021.149834 34525746 PMC8379898

[28] Full Article: History of Impactors—The First 110 Years. Available from: https://www.tandfonline.com/doi/full/10.1080/02786820490424347

[29] Zhou J, Wu J, Zeng X, Huang G, Zou L, Song Y, Gopinath D, Zhang X, Kang M, Lin J, Cowling BJ, Lindsley WG, Ke C, Peiris JSM, Yen H-L. 2016. Isolation of H5N6, H7N9 and H9N2 avian influenza A viruses from air sampled at live poultry markets in China, 2014 and 2015. Euro Surveill 21:30331. doi:10.2807/1560-7917.ES.2016.21.35.30331 27608369 PMC5015459

[30] Horwood PF, Horm SV, Yann S, Tok S, Chan M, Suttie A, Y P, Rith S, Siegers JY, San S, Davun H, Tum S, Ly S, Tarantola A, Dussart P, Karlsson EA. 2023. Aerosol exposure of live bird market workers to viable influenza A/ H5N1 and A/ H9N2 viruses, Cambodia. Zoonoses Public Health 70:171–175. doi:10.1111/zph.13009 36409285 PMC10098856

[31] Cheng KL, Wu J, Shen WL, Wong AYL, Guo Q, Yu J, Zhuang X, Su W, Song T, Peiris M, Yen H-L, Lau EHY. 2020. Avian influenza virus detection rates in poultry and environment at live poultry markets, Guangdong, China. Emerg Infect Dis 26:591–595. doi:10.3201/eid2603.190888 31922954 PMC7045814

[32] Venter JC, Remington K, Heidelberg JF, Halpern AL, Rusch D, Eisen JA, Wu D, Paulsen I, Nelson KE, Nelson W, Fouts DE, Levy S, Knap AH, Lomas MW, Nealson K, White O, Peterson J, Hoffman J, Parsons R, Baden-Tillson H, Pfannkoch C, Rogers Y-H, Smith HO. 2004. Environmental genome shotgun sequencing of the Sargasso Sea. Science 304:66–74. doi:10.1126/science.1093857 15001713

[33] Zhang L, Chen F, Zeng Z, Xu M, Sun F, Yang L, Bi X, Lin Y, Gao Y, Hao H, Yi W, Li M, Xie Y. 2021. Advances in metagenomics and its application in environmental microorganisms. Front Microbiol 12:766364. doi:10.3389/fmicb.2021.766364 34975791 PMC8719654

[34] Watson JD. 1990. The human genome project: past, present, and future. Science 248:44–49. doi:10.1126/science.2181665 2181665

[35] Zhang Y-Z, Chen Y-M, Wang W, Qin X-C, Holmes EC. 2019. Expanding the RNA virosphere by unbiased metagenomics. Annu Rev Virol 6:119–139. doi:10.1146/annurev-virology-092818-015851 31100994

[36] Quince C, Walker AW, Simpson JT, Loman NJ, Segata N. 2017. Shotgun metagenomics, from sampling to analysis. Nat Biotechnol 35:833–844. doi:10.1038/nbt1217-1211b 28898207

[37] Pascher K, Švara V, Jungmeier M. 2022. Environmental DNA-based methods in biodiversity monitoring of protected areas: application range, limitations, and needs. Diversity 14:463. doi:10.3390/d14060463

[38] Batovska J, Mee PT, Lynch SE, Sawbridge TI, Rodoni BC. 2019. Sensitivity and specificity of metatranscriptomics as an arbovirus surveillance tool. Sci Rep 9:19398. doi:10.1038/s41598-019-55741-3 31852942 PMC6920425

[39] Ko KKK, Chng KR, Nagarajan N. 2022. Metagenomics-enabled microbial surveillance. Nat Microbiol 7:486–496. doi:10.1038/s41564-022-01089-w 35365786

[40] Gardy JL, Loman NJ. 2018. Towards a genomics-informed, real-time, global pathogen surveillance system. Nat Rev Genet 19:9–20. doi:10.1038/nrg.2017.88 29129921 PMC7097748

[41] Greninger AL, Chen EC, Sittler T, Scheinerman A, Roubinian N, Yu G, Kim E, Pillai DR, Guyard C, Mazzulli T, Isa P, Arias CF, Hackett J, Schochetman G, Miller S, Tang P, Chiu CY. 2010. A metagenomic analysis of pandemic influenza A (2009 H1N1) infection in patients from North America. PLoS One 5:e13381. doi:10.1371/journal.pone.0013381 20976137 PMC2956640

[42] Rogalski A. 2012. History of infrared detectors. Opto-Electron Rev 20:279–308. doi:10.2478/s11772-012-0037-7

[43] Lisowska-Lis A, Mitkowski SA, Augustyn J. Infrared technique and its application in science and engineering in the study plans of students in electrical engineering and electronics

[44] Don’t Fight the Fever. Available from: https://www.science.org/content/article/dont-fight-fever

[45] Noh J-Y, Kim K-J, Lee S-H, Kim J-B, Kim D-H, Youk S, Song C-S, Nahm S-S. 2021. Thermal image scanning for the early detection of fever induced by highly pathogenic avian influenza virus infection in chickens and ducks and its application in farms. Front Vet Sci 8:616755. doi:10.3389/fvets.2021.616755 34113668 PMC8185153

[46] Nguyen AV, Cohen NJ, Lipman H, Brown CM, Molinari N-A, Jackson WL, Kirking H, Szymanowski P, Wilson TW, Salhi BA, Roberts RR, Stryker DW, Fishbein DB. 2010. Comparison of 3 infrared thermal detection systems and self-report for mass fever screening. Emerg Infect Dis 16:1710–1717. doi:10.3201/eid1611.100703 21029528 PMC3294528

[47] Berckmans D, Hemeryck M, Berckmans D, Vranken E, van T. 2015. Animal sound… talks! real-time sound analysis for health monitoring in livestock. Proc Animal Environment and Welfare:215–222.

[48] Johnson E, Campos-Cerqueira M, Jumail A, Yusni ASA, Salgado-Lynn M, Fornace K. 2023. Applications and advances in acoustic monitoring for infectious disease. Trends Parasitol 39:386–399. doi:10.1016/j.pt.2023.01.008 36842917

[49] Chung Y, Oh S, Lee J, Park D, Chang H-H, Kim S. 2013. Automatic detection and recognition of pig wasting diseases using sound data in audio surveillance systems. Sensors (Basel) 13:12929–12942. doi:10.3390/s131012929 24072029 PMC3859042

[50] Rocha N, Dias A, Santinha G, Rodrigues M, Queirós A, Rodrigues C. 2019. Smart cities and healthcare: asystematic review. Technologies 7:58. doi:10.3390/technologies7030058

[51] Worsley-Tonks KEL, Bender JB, Deem SL, Ferguson AW, Fèvre EM, Martins DJ, Muloi DM, Murray S, Mutinda M, Ogada D, Omondi GP, Prasad S, Wild H, Zimmerman DM, Hassell JM. 2022. Strengthening global health security by improving disease surveillance in remote rural areas of low-income and middle-income countries. Lancet Glob Health 10:e579–e584. doi:10.1016/S2214-109X(22)00031-6 35303467 PMC8923676

[52] Kwok KTT, de Rooij MMT, Messink AB, Wouters IM, Smit LAM, Cotten M, Heederik DJJ, Koopmans MPG, Phan MVT. 2022. Establishing farm dust as a useful viral metagenomic surveillance matrix. Sci Rep 12:16308. doi:10.1038/s41598-022-20701-x 36175536 PMC9521564

[53] de Vries JJC, Brown JR, Fischer N, Sidorov IA, Morfopoulou S, Huang J, Munnink BBO, Sayiner A, Bulgurcu A, Rodriguez C, et al.. 2021. Benchmark of thirteen bioinformatic pipelines for metagenomic virus diagnostics using datasets from clinical samples. J Clin Virol 141:104908. doi:10.1016/j.jcv.2021.104908 34273858 PMC7615111

[54] Qian M, Zhan Y, Wu D, Ji L, Chen H, Cheng Y. 2021. Clinical standardization of metagenomic next generation sequencing (mNGS) in the pathogen diagnosis. Clinical and Translational Dis 1:e12. doi:10.1002/ctd2.12

[55] Sherry NL, Horan KA, Ballard SA, Gonҫalves da Silva A, Gorrie CL, Schultz MB, Stevens K, Valcanis M, Sait ML, Stinear TP, Howden BP, Seemann T. 2023. An ISO-certified genomics workflow for identification and surveillance of antimicrobial resistance. Nat Commun 14:60. doi:10.1038/s41467-022-35713-4 36599823 PMC9813266

[56] Crisan A, McKee G, Munzner T, Gardy JL. 2018. Evidence-based design and evaluation of a whole genome sequencing clinical report for the reference microbiology laboratory. PeerJ 6:e4218. doi:10.7717/peerj.4218 29340235 PMC5767084

[57] Tornheim JA, Starks AM, Rodwell TC, Gardy JL, Walker TM, Cirillo DM, Jayashankar L, Miotto P, Zignol M, Schito M. 2019. Building the framework for standardized clinical laboratory reporting of next-generation sequencing data for resistance-associated mutations in Mycobacterium tuberculosis complex. Clin Infect Dis 69:1631–1633. doi:10.1093/cid/ciz219 30883637 PMC6792097

[58] Bharucha T, Oeser C, Balloux F, Brown JR, Carbo EC, Charlett A, Chiu CY, Claas ECJ, de Goffau MC, de Vries JJC, et al.. 2020. STROBE-metagenomics: a STROBE extension statement to guide the reporting of metagenomics studies. Lancet Infect Dis 20:e251–e260. doi:10.1016/S1473-3099(20)30199-7 32768390 PMC7406238

[59] Jones KE, Patel NG, Levy MA, Storeygard A, Balk D, Gittleman JL, Daszak P. 2008. Global trends in emerging infectious diseases. Nature 451:990–993. doi:10.1038/nature06536 18288193 PMC5960580

[60] Leifels M, Khalilur Rahman O, Sam I-C, Cheng D, Chua FJD, Nainani D, Kim SY, Ng WJ, Kwok WC, Sirikanchana K, Wuertz S, Thompson J, Chan YF. 2022. The one health perspective to improve environmental surveillance of zoonotic viruses: lessons from COVID-19 and outlook beyond. ISME Commun 2:107. doi:10.1038/s43705-022-00191-8 36338866 PMC9618154

[61] Karesh WB, Dobson A, Lloyd-Smith JO, Lubroth J, Dixon MA, Bennett M, Aldrich S, Harrington T, Formenty P, Loh EH, Machalaba CC, Thomas MJ, Heymann DL. 2012. Ecology of zoonoses: natural and unnatural histories. Lancet 380:1936–1945. doi:10.1016/S0140-6736(12)61678-X 23200502 PMC7138068

